# Crystal structure, PIXEL calculations of inter­molecular inter­action energies and solid-state characterization of the herbicide isoxaflutole

**DOI:** 10.1107/S2056989022008647

**Published:** 2022-09-06

**Authors:** Jascha Schinke, Thomas Gelbrich, Ulrich J. Griesser

**Affiliations:** a University of Innsbruck, Institute of Pharmacy, Innrain 52, 6020 Innsbruck, Austria; Vienna University of Technology, Austria

**Keywords:** crystal structure, herbicide, PIXEL calculations

## Abstract

In the isoxaflutole mol­ecule {systematic name: (5-cyclo­propyl-1,2-oxazol-4-yl)[2-(methyl­sulfon­yl)-4-(tri­fluoro­meth­yl)phen­yl]methanone; C_15_H_12_F_3_NO_4_S}, the 1,2-oxazole and methanone fragments form an almost coplanar unit, whereas the methanone and phenyl mean planes are inclined by an angle of more than 60°. This conformation differs fundamentally from all other known examples of the 1,2-oxazol-4-yl(phen­yl)methanone fragment and is ascribed to the presence of the bulky methyl­sulfonyl *para* substituent at the phenyl ring. PIXEL calculations reveal that the largest contributions to the stabilization of the crystal persist within a columnar arrangement of mol­ecules along the twofold screw axis and in inter­actions between adjacent columns related by an inversion operation. Both these intra-column and inter-column motifs are dominated by the dispersion energy term but also display additional significant stabilization effects as a result of three short inter­molecular C—H⋯O contacts involving the methane­sulfonyl-O atoms.

## Abstract 2

The packing of isoxaflutole mol­ecules can be rationalized in terms of a hierarchy of inter­action energies within columnar and layer arrangements, in each case dominated by the dispersion energy term.

## 1. Chemical context

The title compound, (I), belongs to the family of isoxazoles and was originally developed by Rhône-Poulenc Agriculture (Cain *et al.*, 1992 ▸). Isoxaflutole is a preemergence herbicide that is used against grasses and broadleaf weeds (Luscombe *et al.*, 1995 ▸). This compound metabolizes briskly in soils and plants by opening the ring of the isoxazole group. A diketo­nitrile derivate is formed in this process, which acts as an inhibitor of 4-hy­droxy­phenyl­pyruvate di­oxy­genase (HPPD) (Pallett *et al.*, 1997 ▸; Roberts *et al.*, 1999 ▸). Isoxaflutole is marketed in the form of suspension concentrate formulations, water-dispersible granules and wettable powders where it is either the sole active ingredient or combined with other herbicides such as flufenacet. 
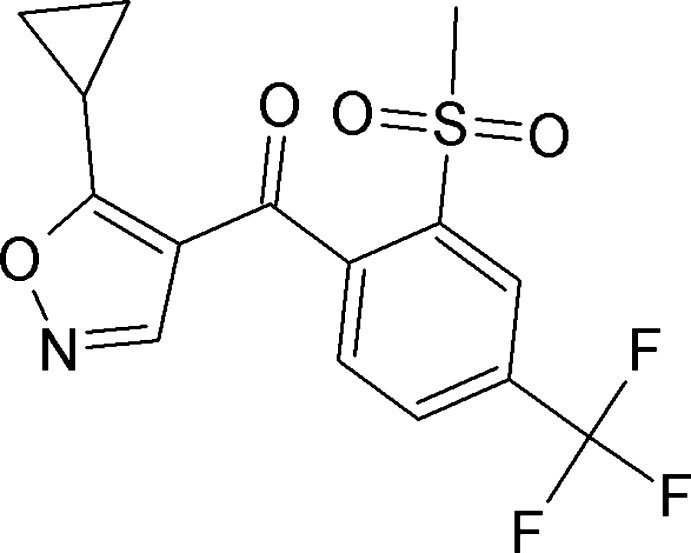


We have studied the solid-state properties of isoxaflutole as part of a wider investigation of herbicides and present the results in the present communication.

## 2. Structural commentary

**Figure 1:**
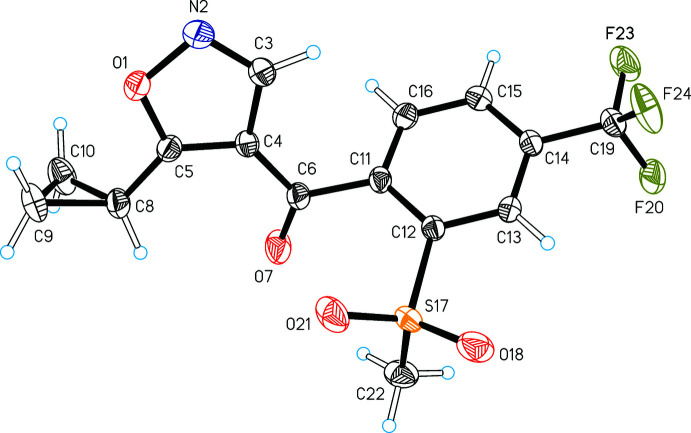
Mol­ecular structure of (I) with displacement ellipsoids drawn at the 50% probability level and hydrogen atoms drawn as spheres of arbitrary size.

The asymmetric unit of (I) contains one mol­ecule (Fig. 1 ▸). The cyclo­propyl substituent (C8, C9, C10) of the oxadazol ring is orientated such that its C8—C9 bond lies approximately parallel to the C5—O1 bond of the ring [torsion angle O1—C5—C8—C9 = −15.0 (3)°]. The methanone fragment (O7, C4, C6, C11) and the oxadazol ring (O1, N2, C3, C4, C5) form an almost planar unit. The angle between their respective mean planes is 4.4 (1)°, and the orientation of the methanone group relative to the cyclo­propyl substituent of the ring is *cis*. By contrast, the methanone mean plane forms an angle of 64.28 (5)° with the phenyl ring (C11–C16). The orientation of the methyl­sulfonyl substituent at the phenyl ring is such that its S17—C22 bond is almost perpendicular to the ring mean plane, which is illustrated by the value of the *pseudo*-torsion angle C15⋯C12—S17—C22 of −83.8°.

## 3. Database survey

**Figure 2:**
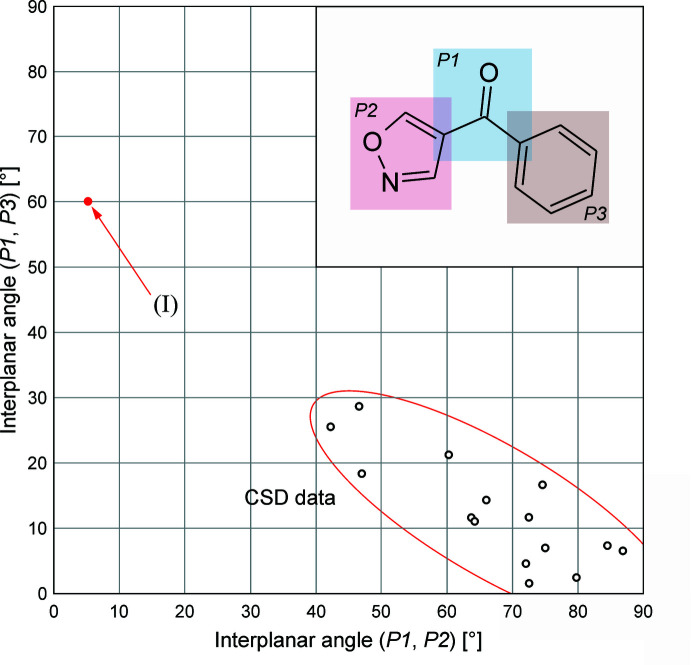
Plot of the inter­planar angles (*P*1, *P*3) against (*P*1, *P*2), illustrating that the isoxaflutole mol­ecule (red circle) has an unusual 1,2-oxazol-4-yl(phen­yl)methanone conformation. Sixteen data points were obtained from 14 CSD structures (open circles) and isoxaflutole (filled red circle; see section 1 of the supporting information).

The Cambridge Structural Database (version 5.43, June 2022; Groom *et al.*, 2016 ▸) contains 15 entries of structures displaying the 1,2-oxazol-4-yl(phen­yl)methanone structure fragment (see Table S1 of the supporting information). The conformation of this structure fragment can be rationalized in terms of the relative orientation of three planar units (see Fig. 2 ▸, inset), *i.e.* the methanone (*P*1), 1,2-oxazole (*P*2) and phenyl (*P*3) fragments. In each of the previous examples, the plane of the methanone fragment tends to approach coplanarity with the phenyl ring. The corresponding inter­planar angle (*P*1, *P*3) ranges between 1.6° and 28.7°. In turn, the methanone and 1,2-oxazole mean planes (*P*1, *P*2) form angles in the range from 42.3° to 86.9°. The diagram in Fig. 2 ▸ illustrates that for a given mol­ecule, a smaller (*P*1, *P*2) angle is generally correlated with a wider (*P*1, *P*3) angle and *vice versa*. Apart from (I), *ortho* substituents at the phenyl ring are present only in DUHKOI (Cl and F; 28.7°/46.6°) and KOQGOM (—OH; 79.8°/2.4°), which displays an intra­molecular O—H⋯O(methanone) bond. The mol­ecules in the sample group have bulky substituents at both the 3- and 5-positions of the 1,2-oxazole ring, except for (I), YELQAK and YELQEO, which have just one such substituent (supporting information, Table S1). The plot of (*P*1, *P*2) against (*P*1, *P*3) angles in Fig. 2 ▸ illustrates the uniqueness of the conformation of (I) with almost coplanar methanone and 1,2-oxazole units (see previous section), whilst the methanone and phenyl rings planes form an angle (*P*1, *P*2) of 64.28 (5)°. This unusual conformation is probably due to the bulky methane­sulfonyl group as an *ortho* substituent of the phenyl ring of (I).

## 4. Supra­molecular features

**Figure 3:**
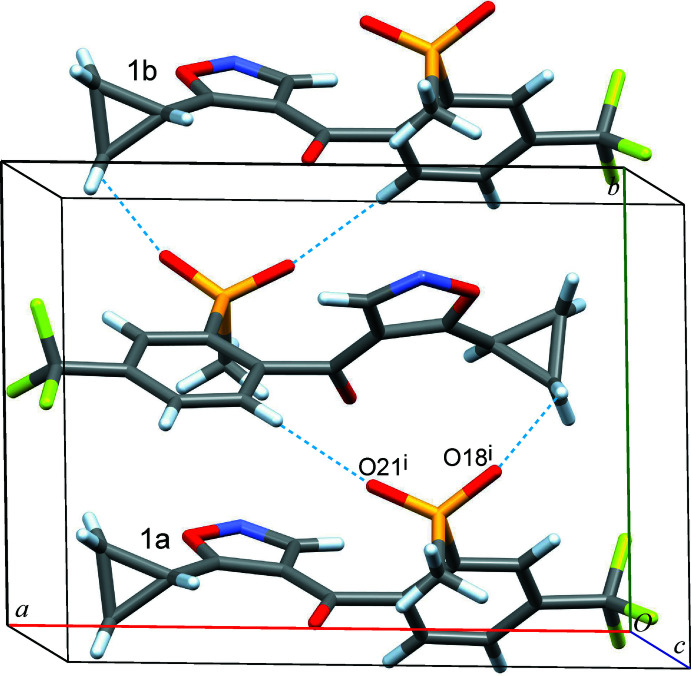
Mol­ecules related by a twofold screw operation form two short C—H⋯O contacts, resulting in a columnar arrangement along the *b* axis (motif 1a,b).

The isoxaflutole mol­ecule does not contain any classical hydrogen-bond donor groups. However, two significant short inter­molecular C—H⋯O contacts are found between mol­ecules related by a twofold screw operation (Table 1 ▸). The first of these, C16—H16⋯O21^i^ involves a CH group of the phenyl ring and a methane­sulfonyl-O atom (H16⋯O21^i^ = 2.33 Å). A somewhat longer C10—H10*A*⋯O18^i^ contact is formed between the other methane­sulfonyl-O atom and the cyclo­propyl group (H10*A*⋯O18^i^ = 2.63 Å). A column-like structure of mol­ecules linked by these contacts propagates parallel to the *b* axis (Fig. 3 ▸). Moreover, columnar structures related by a glide mirror operation of this kind form a layer motif along the *c* axis with short (1,2-oxazol) C3—H3⋯O21^ii^(methane­sulfon­yl) contacts (H3⋯O21^ii^ = 2.71 Å; Table 1 ▸). Parallel stacking of the these supra­molecular *bc* layers in the *a-*axis direction results in multiple F⋯F and F⋯H inter­layer contacts.

## 5. Qu­anti­tative analysis of inter­molecular inter­actions

Inter­molecular inter­action energies were calculated with the semi-classical density sums (SCDS-PIXEL) method using the program *OPiX* (Gavezzotti, 2007 ▸, 2011 ▸). C—H distances were recalculated to standard lengths and an electron-density map was calculated at the MP2/6-31G(d,p) level using *Gaussian09* (Frisch *et al.*, 2009 ▸). The obtained lattice energy of −140 kJ mol^−1^ can be partitioned into contributions from Coulombic (*E*_Col_ = −56.6 kJ mol^−1^), polarization (*E*_pol_ = −20.7 kJ mol^−1^), dispersion (*E*_dis_ = −151.2 kJ mol^−1^) and repulsion (*E*_rep_ = 88.2 kJ mol^−1^) terms. Their relative values indicate that dispersion energy and electrostatic (Coulombic + polarization) energy contribute with 66% and 34%, respectively, to the stabilization of the crystal structure.

Considering the individual inter­action energies computed for pairs of mol­ecules, the largest absolute total contribution by far (*E*_tot_ = −57.2 kJ mol^−1^) is obtained for two symmetry-equivalent inter­actions between a central and two neighbouring mol­ecules related to each other by twofold screw operations (denoted as 1a,b in Table 2 ▸ and Figs. 3 ▸, 4 ▸). The sum of total energies of all mol­ecule/mol­ecule inter­actions in the crystal *E*_tot,S_ is −144.8 kJ mol^−1^, which means that this columnar motif parallel to the *b* axis (see above) alone accounts for approximately 40% of the stabilization of the structure. This arrangement is associated with a large contact area of van der Waals surfaces (*E*_dis_ = −58.7 kJ mol^−1^) and also with significant Coulombic and polarization terms (*E*_Col_ = −28.5 kJ mol^−1^ and *E*_pol_ = −10.1 kJ mol^−1^), which may confirm the attractive nature of the short inter­molecular C16—H16⋯O21^i^ and C10—H10*A*⋯O18^i^ contacts discussed in the previous section (Table 1 ▸, Fig. 3 ▸).

**Figure 4:**
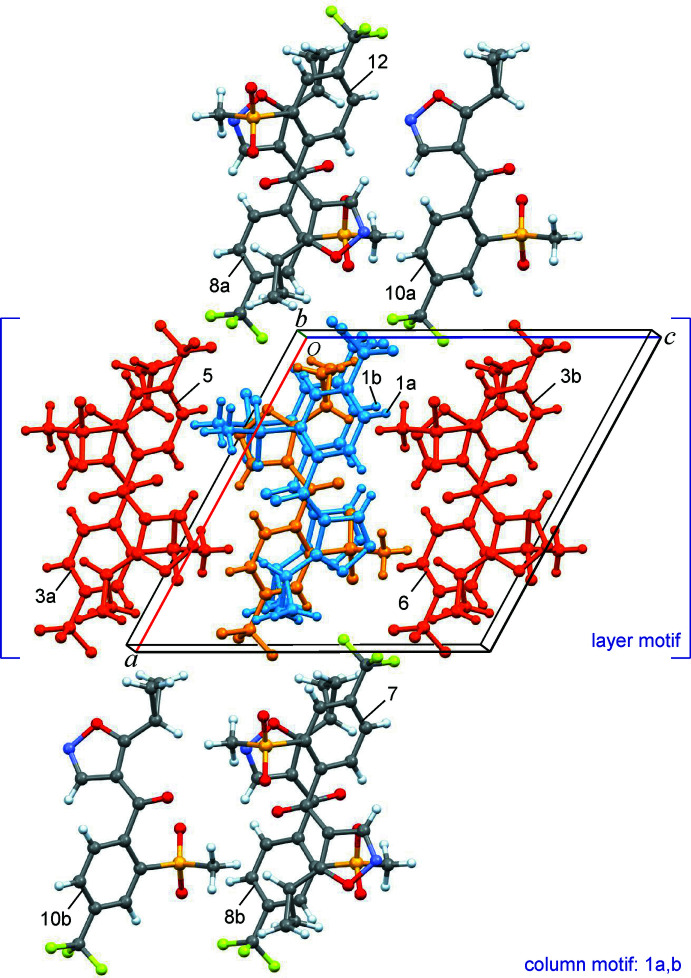
Cluster consisting of a central mol­ecule (orange) and neighbouring mol­ecules representing the twelve most important mol­ecule/mol­ecule inter­actions (see Table 3 ▸). The inter­actions 1a,b (blue mol­ecules) constitute a column along the twofold screw axis, whilst 3a,b, 5 and 6 are inter­actions between adjacent columns related by a *c* glide mirror operation.

Another set of two symmetry-equivalent inter­actions (denoted as 3a,b; *E*_tot_ = −36.7 kJ mol^−1^) are associated with glide mirror operations, *i.e.* the assembly of neighbouring column motifs into a layer structure along the *c* axis. Significant Coulombic (*E*_Col_ = −15.9 kJ mol^−1^) and polarization (*E*_pol_= −7.3 kJ mol^−1^) terms, coinciding with the short C3—H3⋯O21^ii^ contact mentioned in the previous section (Table 1 ▸), are observed in addition to the dominant dispersion energy contributions (*E*_dis_ = −33.4 kJ mol^−1^). The diagram in Fig. 4 ▸ shows a central mol­ecule and its twelve most important mol­ecular neighbours, which together account for approximately 96% of the sum of pairwise PIXEL energies (see section 2 of the supporting information). Altogether, intra-column (along the *b* axis) inter­actions and inter­action between neighbouring columns (along the *c* axis) contribute approximately with 42% and 43%, respectively, to the stabilization of the crystal structure. The rest (15%) originates from the stacking of mol­ecular *bc* layers in the *a-*axis direction.

## 6. Synthesis and crystallization

Isoxaflutole (technical quality) was recrystallized from a hot saturated aceto­nitrile (p.a.) solution yielding a colourless crystalline product used for further characterization. The reported form was also the only crystalline phase encountered in systematic crystallization experiments using a series of solvents (methanol, ethanol, di­chloro­methane, aceto­nitrile, ethyl acetate, acetone, methyl ethyl ketone, tetra­hydro­furan and toluene). Results of further investigations of the crystalline form of (I) comprising hot-stage microscopy, DSC, TGA, ATR–FTIR, Raman spectroscopy and powder X-ray diffraction methods are reported in sections 3 to 7 of the supporting information. In addition, selected data are reported for the amorphous form of (I) obtained by quench cooling the melt to room temperature.

## 7. Refinement

Crystal data, data collection and structure refinement details are summarized in Table 3 ▸. All H atoms were identified in difference-Fourier maps. Methyl H atoms were idealized and included as rigid groups allowed to rotate but not tip (C—H = 0.98 Å). H atoms bonded to aromatic CH (C—H = 0.95 Å), secondary CH_2_ and tertiary CH carbon atoms (C—H = 0.99 Å) were positioned geometrically. The *U*_iso_ parameters of all H atoms were refined freely. Two outlier reflections (102, 202) were omitted from the final refinement.

## Supplementary Material

Crystal structure: contains datablock(s) I. DOI: 10.1107/S2056989022008647/wm5659sup1.cif

Structure factors: contains datablock(s) I. DOI: 10.1107/S2056989022008647/wm5659Isup2.hkl

Supporting information file. DOI: 10.1107/S2056989022008647/wm5659Isup3.mol

Details of the PIXEL calculation and hot-stage microscopy, DSC, TGA, spectroscopic (ATR-FTIR, Raman) and powder X-ray diffraction data. DOI: 10.1107/S2056989022008647/wm5659sup4.pdf

Supporting information file. DOI: 10.1107/S2056989022008647/wm5659Isup5.cml

CCDC reference: 2204082

Additional supporting information:  crystallographic information; 3D view; checkCIF report

## Figures and Tables

**Table 1:** Hydrogen-bond geometry (Å, °)

*D*—H⋯*A*	*D*—H	H⋯*A*	*D*⋯*A*	*D*—H⋯*A*
C16—H16⋯O21^i^	0.95	2.35	3.214 (2)	151
C10—H10*A*⋯O18^i^	0.99	2.63	3.355 (2)	130
C3—H3⋯O21^ii^	0.95	2.71	3.522 (2)	144

Symmetry codes: (i) 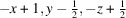 ; (ii) 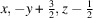 .

**Table 2:** PIXEL energies (kJ mol^−1^) for mol­ecule/mol­ecule inter­actions

Index	Symmetry operations	Symmetry element	*d* (Å)	*E*_Col_	*E*_pol_	*E*_energy-dispersive_	*E*_rep_	*E*_tot_	Motif	Inter­actions
1a,b	1 − *x*, − 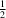 + *y*, 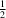 − *z*; 1 − *x*, 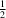 + *y*, 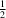 − *z*,	2_1_	5.367	–28.5	–10.1	–58.7	40.0	–57.2	column	C16—H16⋯O21^i^; C10—H10*A*⋯O18^i^
3a,b	*x*, 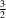 − *y*, − 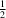 + *z*; *x*, 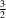 − *y*, 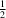 + *z*	*c*	7.002	–15.9	–7.1	–33.4	18.7	–37.6	layer	C3—H3⋯O21^ii^
5	1 − *x*, 1 − *y*, −*z*	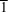	7.261	–9.8	–6.3	–26.0	17.0	–25.2	layer	
6	1 − *x*, 1 − *y*, 1 − *z*	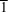	8.141	–6.3	–2.5	–21.5	10.3	–19.9	layer	
7	2 − *x*, 1 − *y*, 1 − *z*	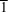	9.686	–5.8	–1.2	–14.5	8.1	–13.4	stack	
8a,b	*x* − 1, 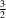 − *y*, − 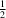 + *z*; 1 + *x*, 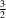 − *y*, 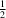 + *z*	*c*	12.086	–0.6	–1.0	–9.7	3.2	–8.1	stack	
10*a*,b	*x* − 1, *y*, *z*; *x* + 1, *y*, *z*	1	13.569	–2.2	–0.8	–6.4	5.0	–4.3	stack	
12	−*x*, 1 − *y*, −*z*	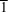	14.807	–1.8	–0.8	–7.4	5.9	–4.1	stack	

**Table 3:** Experimental details

Crystal data	
Chemical formula	C_15_H_12_F_3_NO_4_S
*M*_r_	359.32
Crystal system, space group	Monoclinic, *P*2_1_/*c*
Temperature (K)	193
*a*, *b*, *c* (Å)	13.5689 (16), 9.2906 (8), 13.4358 (15)
β (°)	118.530 (15)
*V* (Å^3^)	1488.1 (3)
*Z*	4
Radiation type	Mo *K*α
μ (mm^−1^)	0.27
Crystal size (mm)	0.25 × 0.10 × 0.08

Data collection	
Diffractometer	Xcalibur, Ruby, Gemini ultra
Absorption correction	Multi-scan (*CrysAlis PRO*; Rigaku OD, 2020 ▸)
*T*_min_, *T*_max_	0.925, 1.000
No. of measured, independent and observed [*I* > 2σ(*I*)] reflections	9962, 3275, 2667
*R*_int_	0.036
(sin θ/λ)_max_ (Å^−1^)	0.641

Refinement	
*R*[*F* ^2^ > 2σ(*F* ^2^)], *wR*(*F* ^2^), *S*	0.038, 0.097, 1.02
No. of reflections	3275
No. of parameters	231
H-atom treatment	Only H-atom displacement parameters refined
Δρ_max_, Δρ_min_ (e Å^−3^)	0.31, −0.35

Computer programs: *CrysAlis PRO* (Rigaku OD, 2020 ▸), *SHELXT* (Sheldrick, 2015*a* ▸), *SHELXL* (Sheldrick, 2015*b* ▸), *XP* (Bruker, 1998 ▸), *Mercury* (Macrae *et al.*, 2020 ▸), *PLATON* (Spek, 2020 ▸) and *publCIF* Westrip (2010 ▸).

## supplementary crystallographic information

### Crystal data

**Table:**

C_15_H_12_F_3_NO_4_S	*F*(000) = 736
*M**_r_* = 359.32	*D*_x_ = 1.604 Mg m^−^^3^
Monoclinic, *P*2_1_/*c*	Mo *K*α radiation, λ = 0.71073 Å
*a* = 13.5689 (16) Å	Cell parameters from 2902 reflections
*b* = 9.2906 (8) Å	θ = 5.0–29.8°
*c* = 13.4358 (15) Å	µ = 0.27 mm^−^^1^
β = 118.530 (15)°	*T* = 193 K
*V* = 1488.1 (3) Å^3^	Prism, colourless
*Z* = 4	0.25 × 0.10 × 0.08 mm

### Data collection

**Table:**

Xcalibur, Ruby, Gemini ultra diffractometer	3275 independent reflections
Radiation source: fine-focus sealed X-ray tube, Enhance (Mo) X-ray Source	2667 reflections with *I* > 2σ(*I*)
Graphite monochromator	*R*_int_ = 0.036
Detector resolution: 10.3575 pixels mm^-1^	θ_max_ = 27.1°, θ_min_ = 2.8°
ω scans	*h* = −17→16
Absorption correction: multi-scan (CrysAlisPro; Rigaku OD, 2020)	*k* = −11→9
*T*_min_ = 0.925, *T*_max_ = 1.000	*l* = −17→17
9962 measured reflections	

### Refinement

**Table:**

Refinement on *F*^2^	Secondary atom site location: difference Fourier map
Least-squares matrix: full	Hydrogen site location: inferred from neighbouring sites
*R*[*F*^2^ > 2σ(*F*^2^)] = 0.038	Only H-atom displacement parameters refined
*wR*(*F*^2^) = 0.097	*w* = 1/[σ^2^(*F*_o_^2^) + (0.0428*P*)^2^ + 0.6729*P*] where *P* = (*F*_o_^2^ + 2*F*_c_^2^)/3
*S* = 1.02	(Δ/σ)_max_ = 0.001
3275 reflections	Δρ_max_ = 0.31 e Å^−^^3^
231 parameters	Δρ_min_ = −0.35 e Å^−^^3^
0 restraints	Extinction correction: SHELXL, Fc^*^=kFc[1+0.001xFc^2^λ^3^/sin(2θ)]^-1/4^
Primary atom site location: structure-invariant direct methods	Extinction coefficient: 0.0203 (15)

### Special details

**Table:**

Geometry. All esds (except the esd in the dihedral angle between two l.s. planes) are estimated using the full covariance matrix. The cell esds are taken into account individually in the estimation of esds in distances, angles and torsion angles; correlations between esds in cell parameters are only used when they are defined by crystal symmetry. An approximate (isotropic) treatment of cell esds is used for estimating esds involving l.s. planes.

### Fractional atomic coordinates and isotropic or equivalent isotropic displacement parameters (Å^2^)

**Table:**

	*x*	*y*	*z*	*U*_iso_*/*U*_eq_	
O1	0.24820 (10)	0.73141 (14)	0.02968 (10)	0.0294 (3)	
N2	0.32693 (13)	0.75772 (18)	−0.01032 (12)	0.0306 (4)	
C3	0.42144 (15)	0.7107 (2)	0.06926 (14)	0.0258 (4)	
H3	0.4898	0.7158	0.0662	0.033 (5)*	
C4	0.41253 (14)	0.65039 (18)	0.16220 (13)	0.0217 (4)	
C5	0.30138 (14)	0.66771 (19)	0.13150 (13)	0.0225 (4)	
C6	0.49824 (14)	0.58226 (19)	0.26500 (14)	0.0242 (4)	
O7	0.47773 (11)	0.52442 (17)	0.33418 (11)	0.0398 (4)	
C8	0.23389 (15)	0.6311 (2)	0.18562 (15)	0.0283 (4)	
H8	0.2763	0.6121	0.2689	0.051 (6)*	
C9	0.11929 (16)	0.6956 (2)	0.14281 (18)	0.0364 (5)	
H9A	0.0941	0.7183	0.1991	0.047 (6)*	
H9B	0.0930	0.7637	0.0785	0.061 (8)*	
C10	0.12928 (17)	0.5426 (2)	0.11990 (19)	0.0381 (5)	
H10A	0.1094	0.5152	0.0413	0.050 (7)*	
H10B	0.1105	0.4698	0.1620	0.059 (7)*	
C11	0.61644 (14)	0.58208 (18)	0.28108 (13)	0.0216 (4)	
C12	0.70654 (13)	0.65149 (18)	0.37110 (13)	0.0193 (3)	
C13	0.81286 (13)	0.64965 (18)	0.38042 (13)	0.0203 (3)	
H13	0.8729	0.6992	0.4408	0.024 (5)*	
C14	0.83109 (14)	0.57524 (18)	0.30129 (13)	0.0206 (3)	
C15	0.74423 (14)	0.50231 (18)	0.21388 (14)	0.0234 (4)	
H15	0.7574	0.4491	0.1610	0.035 (5)*	
C16	0.63762 (14)	0.50723 (19)	0.20371 (14)	0.0255 (4)	
H16	0.5778	0.4584	0.1426	0.031 (5)*	
S17	0.69237 (4)	0.74778 (5)	0.47805 (3)	0.02312 (14)	
O18	0.78878 (12)	0.83833 (16)	0.53321 (11)	0.0387 (4)	
C19	0.94598 (15)	0.5802 (2)	0.31081 (14)	0.0258 (4)	
F20	1.02531 (9)	0.53642 (16)	0.41237 (10)	0.0490 (4)	
O21	0.58332 (12)	0.81258 (15)	0.42868 (11)	0.0375 (4)	
C22	0.69982 (18)	0.6145 (2)	0.57370 (15)	0.0336 (4)	
H22A	0.7714	0.5632	0.6026	0.048 (7)*	
H22B	0.6378	0.5465	0.5351	0.057 (7)*	
H22C	0.6945	0.6594	0.6370	0.046 (6)*	
F23	0.95559 (9)	0.49764 (13)	0.23536 (10)	0.0394 (3)	
F24	0.97390 (10)	0.71318 (13)	0.29720 (12)	0.0467 (3)	

### Atomic displacement parameters (Å^2^)

**Table:**

	*U*^11^	*U*^22^	*U*^33^	*U*^12^	*U*^13^	*U*^23^
O1	0.0211 (7)	0.0400 (8)	0.0249 (6)	0.0034 (5)	0.0093 (5)	0.0067 (5)
N2	0.0295 (9)	0.0387 (10)	0.0257 (7)	−0.0010 (7)	0.0150 (7)	0.0041 (6)
C3	0.0232 (9)	0.0302 (10)	0.0261 (8)	−0.0031 (7)	0.0133 (7)	0.0004 (7)
C4	0.0185 (8)	0.0246 (9)	0.0229 (8)	−0.0039 (7)	0.0106 (7)	−0.0007 (7)
C5	0.0198 (8)	0.0243 (9)	0.0214 (8)	−0.0009 (7)	0.0081 (7)	0.0002 (7)
C6	0.0188 (8)	0.0295 (10)	0.0249 (8)	−0.0023 (7)	0.0110 (7)	0.0016 (7)
O7	0.0245 (7)	0.0600 (10)	0.0369 (7)	0.0013 (7)	0.0162 (6)	0.0207 (7)
C8	0.0191 (9)	0.0374 (11)	0.0299 (9)	−0.0001 (8)	0.0129 (7)	0.0032 (8)
C9	0.0270 (10)	0.0378 (11)	0.0528 (12)	0.0047 (9)	0.0259 (9)	0.0006 (9)
C10	0.0296 (11)	0.0376 (11)	0.0547 (12)	−0.0076 (9)	0.0263 (10)	−0.0072 (9)
C11	0.0176 (8)	0.0243 (9)	0.0230 (8)	0.0003 (7)	0.0099 (7)	0.0054 (6)
C12	0.0181 (8)	0.0202 (8)	0.0207 (7)	0.0016 (6)	0.0100 (6)	0.0020 (6)
C13	0.0171 (8)	0.0213 (8)	0.0207 (8)	−0.0003 (6)	0.0076 (6)	0.0004 (6)
C14	0.0193 (8)	0.0210 (8)	0.0235 (8)	0.0024 (6)	0.0118 (7)	0.0042 (6)
C15	0.0263 (9)	0.0231 (9)	0.0235 (8)	0.0000 (7)	0.0140 (7)	−0.0008 (7)
C16	0.0227 (9)	0.0281 (10)	0.0240 (8)	−0.0061 (7)	0.0097 (7)	−0.0044 (7)
S17	0.0255 (3)	0.0244 (2)	0.0244 (2)	0.00307 (17)	0.01593 (19)	0.00053 (16)
O18	0.0450 (9)	0.0421 (8)	0.0388 (7)	−0.0153 (7)	0.0280 (7)	−0.0174 (6)
C19	0.0238 (9)	0.0284 (10)	0.0296 (9)	0.0024 (7)	0.0164 (7)	0.0029 (7)
F20	0.0193 (6)	0.0889 (10)	0.0363 (6)	0.0101 (6)	0.0112 (5)	0.0143 (6)
O21	0.0378 (8)	0.0411 (8)	0.0414 (8)	0.0201 (6)	0.0252 (7)	0.0109 (6)
C22	0.0411 (12)	0.0370 (11)	0.0263 (9)	0.0069 (9)	0.0191 (9)	0.0082 (8)
F23	0.0358 (7)	0.0455 (7)	0.0497 (7)	0.0046 (5)	0.0306 (6)	−0.0083 (5)
F24	0.0436 (7)	0.0327 (7)	0.0844 (9)	−0.0068 (6)	0.0471 (7)	0.0000 (6)

### Geometric parameters (Å, º)

**Table:**

O1—C5	1.341 (2)	C11—C12	1.400 (2)
O1—N2	1.4276 (19)	C12—C13	1.387 (2)
N2—C3	1.292 (2)	C12—S17	1.7794 (16)
C3—C4	1.426 (2)	C13—C14	1.386 (2)
C3—H3	0.9500	C13—H13	0.9500
C4—C5	1.370 (2)	C14—C15	1.380 (2)
C4—C6	1.458 (2)	C14—C19	1.503 (2)
C5—C8	1.456 (2)	C15—C16	1.387 (2)
C6—O7	1.215 (2)	C15—H15	0.9500
C6—C11	1.513 (2)	C16—H16	0.9500
C8—C9	1.501 (3)	S17—O18	1.4291 (14)
C8—C10	1.508 (3)	S17—O21	1.4333 (14)
C8—H8	1.0000	S17—C22	1.7521 (18)
C9—C10	1.474 (3)	C19—F23	1.326 (2)
C9—H9A	0.9900	C19—F24	1.330 (2)
C9—H9B	0.9900	C19—F20	1.335 (2)
C10—H10A	0.9900	C22—H22A	0.9800
C10—H10B	0.9900	C22—H22B	0.9800
C11—C16	1.390 (2)	C22—H22C	0.9800
			
C5—O1—N2	108.95 (12)	C12—C11—C6	123.48 (15)
C3—N2—O1	104.84 (13)	C13—C12—C11	120.91 (14)
N2—C3—C4	113.07 (16)	C13—C12—S17	116.24 (12)
N2—C3—H3	123.5	C11—C12—S17	122.85 (12)
C4—C3—H3	123.5	C14—C13—C12	119.72 (15)
C5—C4—C3	103.41 (14)	C14—C13—H13	120.1
C5—C4—C6	126.98 (15)	C12—C13—H13	120.1
C3—C4—C6	129.59 (15)	C15—C14—C13	120.32 (15)
O1—C5—C4	109.72 (14)	C15—C14—C19	121.13 (15)
O1—C5—C8	116.89 (15)	C13—C14—C19	118.51 (15)
C4—C5—C8	133.39 (15)	C14—C15—C16	119.61 (15)
O7—C6—C4	123.08 (16)	C14—C15—H15	120.2
O7—C6—C11	120.32 (15)	C16—C15—H15	120.2
C4—C6—C11	116.57 (13)	C15—C16—C11	121.41 (15)
C5—C8—C9	119.94 (16)	C15—C16—H16	119.3
C5—C8—C10	118.40 (16)	C11—C16—H16	119.3
C9—C8—C10	58.67 (13)	O18—S17—O21	118.66 (9)
C5—C8—H8	115.9	O18—S17—C22	108.52 (10)
C9—C8—H8	115.9	O21—S17—C22	108.75 (9)
C10—C8—H8	115.9	O18—S17—C12	106.83 (8)
C10—C9—C8	60.89 (13)	O21—S17—C12	108.86 (8)
C10—C9—H9A	117.7	C22—S17—C12	104.27 (9)
C8—C9—H9A	117.7	F23—C19—F24	107.07 (14)
C10—C9—H9B	117.7	F23—C19—F20	106.22 (14)
C8—C9—H9B	117.7	F24—C19—F20	106.25 (16)
H9A—C9—H9B	114.8	F23—C19—C14	113.20 (15)
C9—C10—C8	60.44 (13)	F24—C19—C14	111.64 (14)
C9—C10—H10A	117.7	F20—C19—C14	112.02 (13)
C8—C10—H10A	117.7	S17—C22—H22A	109.5
C9—C10—H10B	117.7	S17—C22—H22B	109.5
C8—C10—H10B	117.7	H22A—C22—H22B	109.5
H10A—C10—H10B	114.8	S17—C22—H22C	109.5
C16—C11—C12	117.98 (15)	H22A—C22—H22C	109.5
C16—C11—C6	118.53 (15)	H22B—C22—H22C	109.5

### Hydrogen-bond geometry (Å, º)

**Table:**

*D*—H···*A*	*D*—H	H···*A*	*D*···*A*	*D*—H···*A*
C16—H16···O21^i^	0.95	2.35	3.214 (2)	151
C10—H10*A*···O18^i^	0.99	2.63	3.355 (2)	130
C3—H3···O21^ii^	0.95	2.71	3.522 (2)	144

Symmetry codes: (i) −*x*+1, *y*−1/2, −*z*+1/2; (ii) *x*, −*y*+3/2, *z*−1/2.
