# Supplementary material for: Crystal structure, PIXEL calculations of inter­molecular inter­action energies and solid-state characterization of the herbicide isoxaflutole
Source: Acta Crystallogr E Crystallogr Commun. 2022 Sep 6;78(Pt 10):979–83. doi: 10.1107/S2056989022008647 (PMC9535835; doi:10.1107/S2056989022008647)
Supplement: Supplementary file 4 [file e-78-00979-sup4.pdf]

Supporting Information

Crystal structure, PIXEL calculations of intermolecular interaction energies and solid-state characterization of the herbicide isoxaflutole

Jascha Schinke, Thomas Gelbrich* and Ulrich Griesser

Institute of Pharmacy, University of Innsbruck, Innrain 52, 6020 Innsbruck, Austria.

thomas.gelbrich@uibk.ac.at

Contents

1. Conformational analysis 3
2. PIXEL calculations 4
3. Hot-stage microscopy (HSM) 6
4. Differential scanning calorimetry (DSC) and thermogravimetric analysis (TGA) 8
5. ATR-FTIR spectroscopy 9
6. Raman spectroscopy 10
7. Powder X-ray diffraction (PXRD) 11
8. References 12

1. Conformational analysis

The search of the Cambridge Structural Database (version 5.43, June 2022) (Groom *et al.*, 2016) for the 1,2-oxazol-4-yl(phenyl)methanone fragment yielded the 15 entries listed in Table S1 (16 structure fragments).

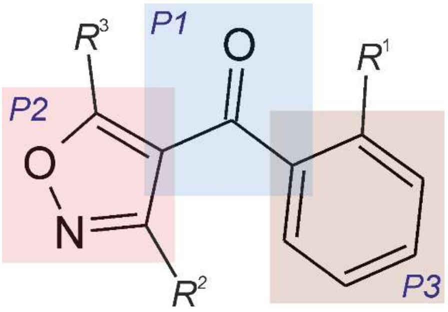

Fig. S1. Definition of the planes *P*1, *P*2 and *P*3 characterising the conformation of 1,2-oxazol-4-yl(phenyl)methanone.

Table S1. Parameters of 1,2-oxazol-4-yl(phenyl)methanone fragments contained in the CSD: interplanar angles and the presence (+) or absence (–) of an *ortho* substituent R^1 at the phenyl ring and substituents *R*2 and *R*3 at the 3- and 5-positions of the 1,2-oxazole ring (see Fig. S1).

CSD refcode	∠(*P*1,*P*2) (°)	∠(*P*1,*P*3) (°)	R^1	R^2	R^3
DUHKOI	46.6	28.7	Cl, F	+	+
IVOQIV	74.6	16.6	–	+	+
KOQGOM	79.8	2.4	OH [b]	+	+
NEHYEG	72.1	4.6	–	+	+
NEHYIK	84.5	7.3	–	+	+
OMETUX	66.0	14.3	–	+	+
OMETUX	60.3	21.3	–	+	+
OMEVAF	72.5	11.7	–	+	+
OMEVEJ	42.3	25.5	–	+	+
OMEVIN	64.3	11.1	–	+	+
OMEVOT	75.1	7.0	–	+	+
QODFIZ	72.6	1.6	–	+	+
YELQAK	63.8	11.6	–	–	+
YELQEO	47.0	18.4	–	+	–
YODRUH	86.9	6.5	–	+	+
(I) [a]	5.2	60.1	$-S(O_2)CH_3$	–	+

[a] This work. [b] Formation of an intermolecular O–H···O(methanone) bond.

2. PIXEL calculations

Energy contributions from the twelve most important molecule/molecule interactions in the crystal structure of isoxaflutole are listed in Table S2 and visualized in Fig. S2. The total PIXEL energy of the lattice is, $E_{tot,Cry}$ = –140.3 kJ mol^{-1}, can be partitioned into contributions from Coulombic (E_{Col} = -56.6 kJ mol^{-1}), polarization (E_{pol} = -20.7 kJ mol^{-1}), dispersion (E_{dis} = -151.2 kJ mol^{-1}) and repulsion (E_{rep} = 88.2 kJ mol^{-1}) terms. To evaluate the relative contribution from certain structure motifs, such as a column of molecules along the *b* axis or a molecular layer parallel the *bc* plane which results from the stacking of such columns along the *c* axis via inversion operations, sums of total energy contributions from all involved interactions $\Sigma^i E_{tot}$ were calculated and compared to the sum of all total energy contributions in the crystal $E_{tot,\Sigma}$ = –144.8 kJ mol^{-1}. (There is small difference between $E_{tot,Cry}$ and $E_{tot,\Sigma}$ as PIXEL energies are not mutually additive.)

Table S2. Pairwise interaction energies (kJ mol^{-1}) for (i = index of an interaction, d = distance between the centroids of two molecules, E_{Col} = Coulombic energy, E_{pol} = polarization energy, E_{dis} = dispersion energy, E_{rep} = repulsion energy, E_{tot} = total PIXEL energy, $\Sigma^i E_{tot}$ = sum of the fist *i* total PIXEL energies and $\Sigma^i E_{tot}$ / $E_{tot,\Sigma}$ is their relative contribution to the sum of all total PIXEL energies).

i	Symmetry operation	*d* (Å)	E_{Col}	E_{pol}	E_{disp}	E_{rep}	E_{tot}	$\Sigma^i E_{tot}$	$\Sigma^i E_{tot}$ / $E_{tot,\Sigma}$	Type
1a	1–*x*, –1/2+*y*, 1/2–*z*	5.367	–28.5	–10.1	–58.7	40.0	–57.2	–28.6	19.8%	Intra-column
1b	1–*x*, 1/2+*y*, 1/2–*z*	5.367	–28.5	–10.1	–58.7	40.0	–57.2	–57.2	39.5%	Intra-column
3a	*x*, 3/2–*y*, –1/2+*z*	7.002	–15.9	–7.1	–33.4	18.7	–37.6	–76.0	52.5%	Intra-plane
3b	*x*, 3/2–*y*, 1/2+*z*	7.002	–15.9	–7.1	–33.4	18.7	–37.6	–94.8	65.5%	Intra-plane
5	1–*x*, 1–*y*, –*z*	7.261	–9.8	–6.3	–26.0	17.0	–25.2	–107.4	74.2%	Stack
6	1–*x*, 1–*y*, 1–*z*	8.141	–6.3	–2.5	–21.5	10.3	–19.9	–117.4	81.1%	Stack
7	2–*x*, 1–*y*, 1–*z*	9.686	–5.8	–1.2	–14.5	8.1	–13.4	–124.1	85.7%	Stack
8a	*x*–1, 3/2–*y*, -1/2+*z*	12.086	–0.6	–1.0	–9.7	3.2	–8.1	–128.1	88.5%	Stack
8b	1+*x*, 3/2–*y*, 1/2+*z*	12.086	–0.6	–1.0	–9.7	3.2	–8.1	–132.2	91.3%	Stack
10a	*x*–1, *y*, *z*	13.569	–2.2	–0.8	–6.4	5.0	–4.3	–134.3	92.8%	Stack
10b	*x*+1, *y*, *z*	13.569	–2.2	–0.8	–6.4	5.0	–4.3	–136.5	94.3%	Stack
12	–*x*, 1–*y*, –*z*	14.807	–1.8	–0.8	–7.4	5.9	–4.1	–138.5	95.7%	Stack

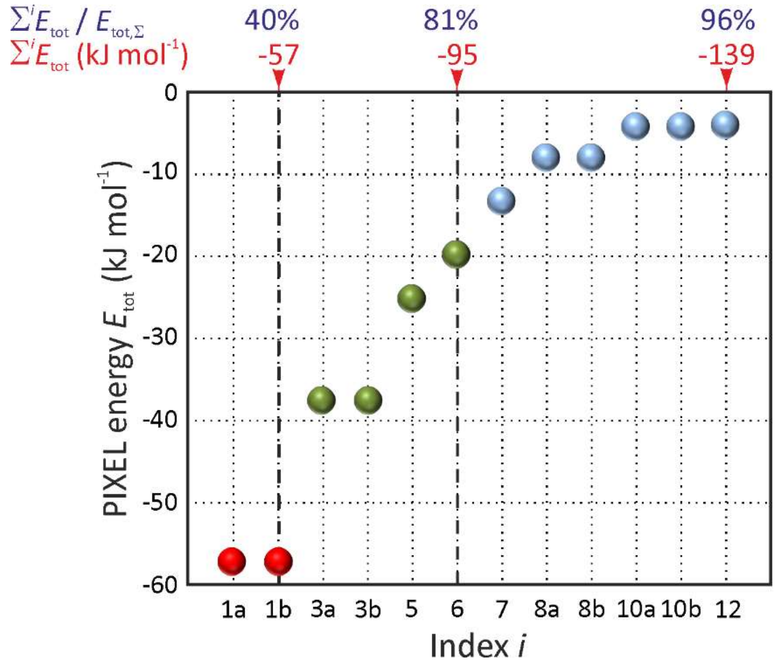

Fig. S2. Visualization of total energy contributions from the first 12 molecule/molecule interactions in the crystal structure of isoxaflutole. Interaction energy contributions are represented as red (intra-columnar), green (inter-columnar along the *c* axis) or gray (stacking of *bc* planes along the axis). $\sum^{i} \boldsymbol{E}_{\mathbf{tot}}$ is the sum of total PIXEL energies of the first *i* interactions and $E_{tot,\Sigma}$ is the sum of the total PIXEL energies.

3. Hot-stage microscopy (HSM)

The prismatic crystals obtained from acetonitrile (

Fig. S3a) show sublimation to acicular crystals depositing on the cover slip (

Fig. S3c) from 120 °C and condensation droplets appear above 130 °C (

Fig. S3b and S3c). The crystals start to melt at 140 °C

Fig. S3b and are in equilibrium with the melt at 140.5 °C. From around 120 °C residual crystals in the melt grow on cooling to flat prisms. The clear melt does not recrystallize on slowly or fast cooling to room temperature nor on reheating indicating a low primary nucleation tendency of isoxaflutole crystals in the melt. Crystallization of the melt can be induced by shear stress or requires seeding. The highest growth rate of crystals in the melt is observed between 110 and 120 °C (Fig. S4).

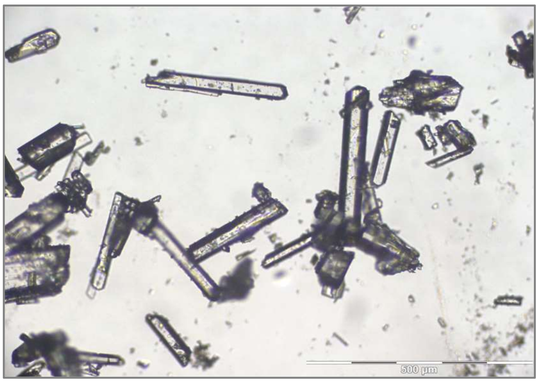

a) Crystals as obtained from acetonitrile (25 °C)

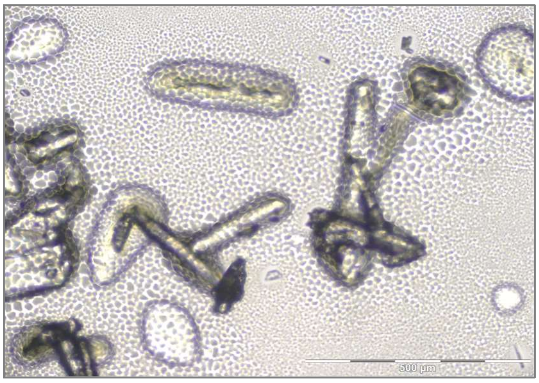

b) T =140.0 °C (melting process starts)

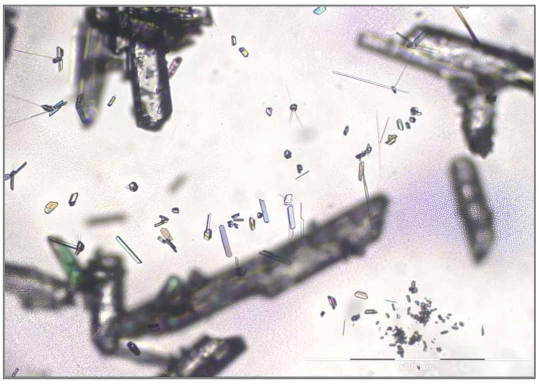

c) Sublimates and condensation droplets occurring close to the melting point at the lower surface of the cover slip

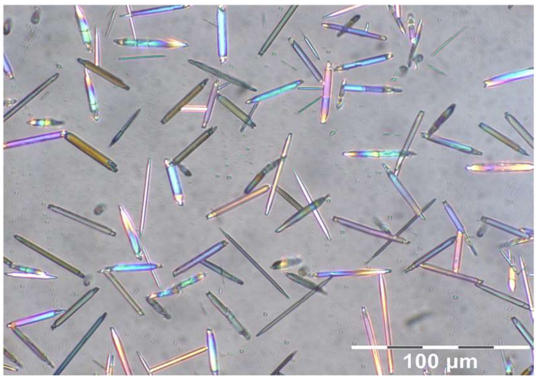

d) Acicular sublimates deposited on the lower surface of the glass bridge

Fig. S3. Microscopic images of isoxaflutole (HSM).

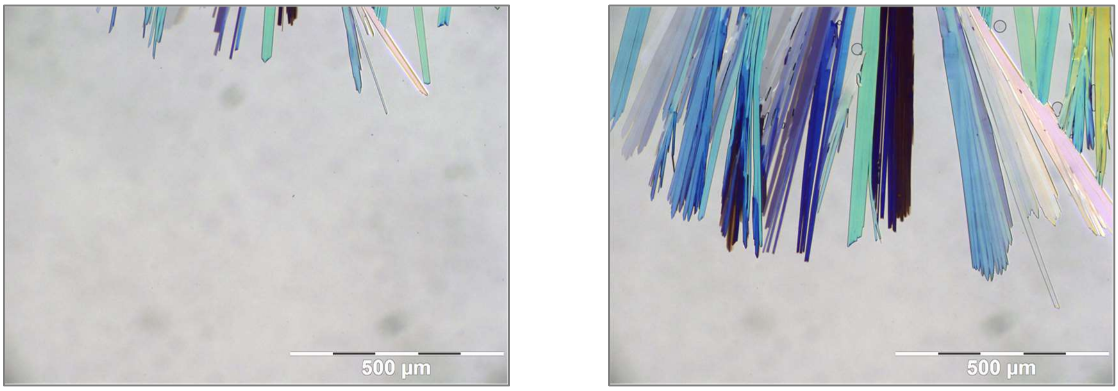

T = 115 °C; t = 0 s T = 115 °C; t = 60 s

Fig. S4. HSM-micrographs of isoxaflutole, illustrating the growth rate at 115 °C .

Experimental: Hot stage microscopy investigations (HSM) were performed with a Reichert Thermovar® polarization microscope (Reichert, Vienna, A) equipped with a Kofler hot stage (Reichert, Vienna, A). The temperature was measured with a digital thermometer (Fluke 51 II) and the temperature calibration was performed with WHO melting point standards.

4. Differential scanning calorimetry (DSC) and thermogravimetric analysis (TGA)

DSC recordings of isoxaflutole show only one sharp melting endotherm at 140.3 °C (Fig. S5) with an enthalpy of fusion of 34.2 kJ mol^{-1}. The TGA starts to slightly decrease from about 120 °C which indicates sublimation and is consistent with thermomicroscopic observations. Above 150 °C the mass loss rate due to evaporation increases quickly.

Thermochemical data obtained for isoxaflutole are collected in Table S3. A melting point of 140 °C has been reported previously,[1] which is consistent with the results of this study.

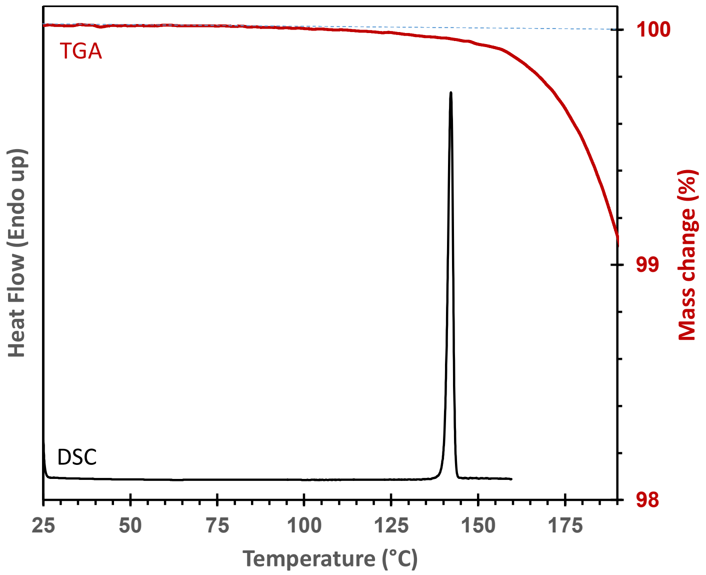

Fig. S5. DSC (sealed pn) and TGA traces of isoxaflutole (heating rate: 10 K min^{-1}).

Table S3. Thermoanalytical data of isoxaflutole obtained in the present study.

**Parameter**	**Value**
T_{fus} (°C) ^{a}	
DSC (onset)	140.3 ± 0.1
HSM (equilibrium)	140.5
$\Delta_{\text{fus}}H$ (kJ mol^{-1}) ^{b}	34.3 ± 0.2
$\Delta_{\text{fus}}S$ (J mol^{-1} K^{-1}) ^{c}	83.0 ± 0.4

^{a} Melting point. ^{b} Enthalpy of fusion. ^{c} Entropy of fusion.

Experimental: *Differential scanning calorimetry* was performed with a DSC 7 (PerkinElmer, Norwalk, Ct., USA). Approximately 1 to 5 ± 0.0005 mg sample (using a UM3 ultramicrobalance, Mettler, Greifensee, CH) were weighed into Al-Pans (30 µL) and sealed with a cover. Dry nitrogen was used as the purge gas (purge: 20 mL min^{-1}). The instrument was calibrated for temperature with pure benzophenone (mp 48.0 °C) and caffeine (236.2 °C), and the energy calibration was performed with indium (mp 156.6 °C, heat of fusion 28.45 J g^{-1}). The errors on the stated temperatures (extrapolated onset temperatures) and enthalpy values were calculated at the 95 % confidence interval (CI) and are based on three measurements. *Thermogravimetric analysis* was carried out with a TGA7 system (Perkin-Elmer) using a sample amount of roughly 5 mg and a platinum sample pan. Temperature calibration was performed with ferromagnetic materials (Alumel and Ni, Curie-point standards, Perkin-Elmer). A heating rate of 5 °C min^{-1} was applied and dry nitrogen was used as a purge gas (sample purge: 20 mL min^{-1}, balance purge: 40 mL min^{-1}).

5. ATR-FTIR spectroscopy

The ATR-FTIR spectra of isoxaflutole (Fig. S6) show a characteristic band at 1667 cm^{-1} (ν C=O) and at 1663 cm^{-1} (ν C=O) for the crystalline and amorphous state, respectively. The obtained peak positions of the crystalline substance match with the bands listed in literature[2] (Table S4).

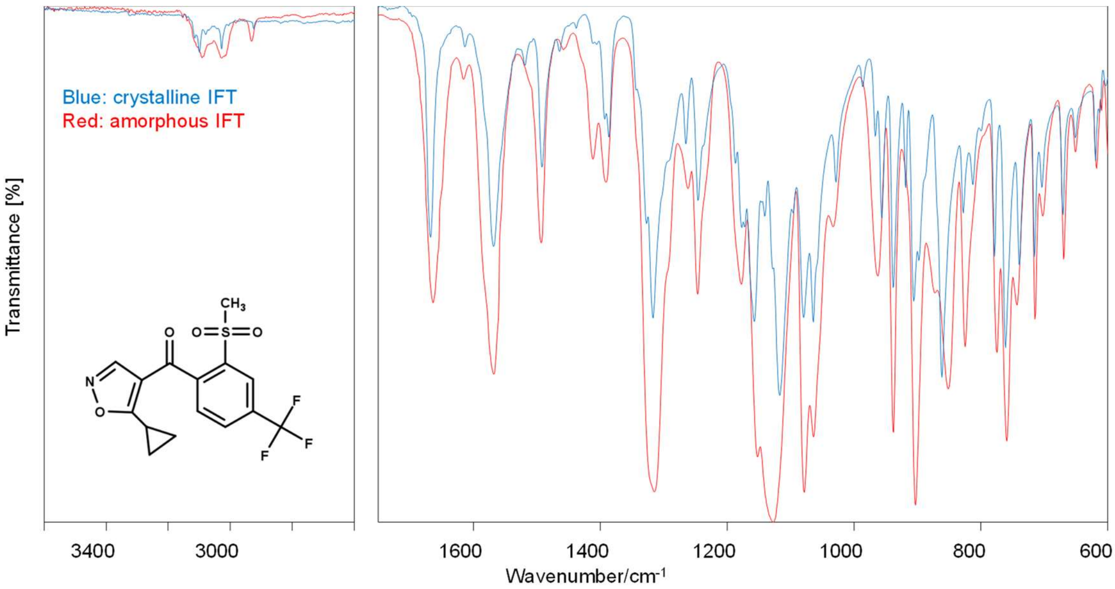

Fig. S6. ATR-FTIR-spectra of isoxaflutole (blue: crystalline; red: amorphous).

Table S4. Comparison of the recorded wavenumbers to literature data.[2]

Wavenumber (cm^{-1}; this work; amorphous)	Wavenumber (cm^{-1}; this work; crystalline)	Wavenumber (cm^{-1}; lit. 2)
3091	3100	3099
3027	3028	3028
1663	1667	1671
1568	1568	1572
1493	1492	1493
1391	1386	1393
1315	1317	1319
1246	1246	1248
1177	1157	1159
1127	1116	1121
1078	1079	1082
962	956	957
938	938	939
903	905	907
851	861	862
775	778	779
759	761	762

Experimental: Infrared spectra were recorded with a diamond ATR unit (PIKE GladiATR, Madison, US) on a Bruker Vertex 70 FTIR spectrometer (Bruker Analytische Messtechnik GmbH, Germany). The spectra were recorded between 4000 and 400 cm^{-1} at an instrument resolution of 2 cm^{-1} (32 scans per spectrum).

6. Raman spectroscopy

The Raman spectra (Fig. S7) of the crystalline and amorphous substance are shown and the Raman bands are compared in Table S5.

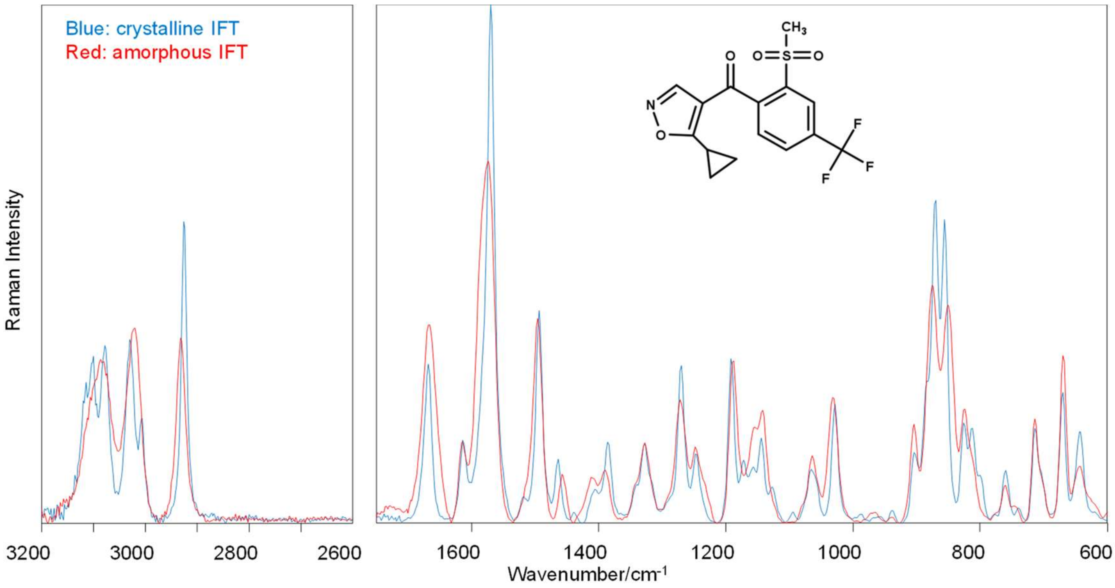

Fig. S7. Raman spectra of isoxaflutole (blue: crystalline; red: amorphous).

Table S5. Characteristic Raman bands for isoxaflutole.

Wavenumber (cm^{-1}; amorphous)	Wavenumber (cm^{-1}; crystalline)
3087	3078
3021	3029
2931	2925
1667	1668
1574	1570
1496	1494
1272	1271
1189	1192
1031	1028
875	871
851	856
669	670

Experimental: Raman spectra were recorded with a Bruker BRAVO hand-held spectrometer (Bruker GmbH, Karlsruhe, D) in the spectral range from 3200 to 300 cm^{-1} with 32 scans and an integration time of 1000 ms per spectrum. The solid samples were prepared on aluminum sample holders.

7. Powder X-ray diffraction (PXRD)

The comparison of the simulated and experimental PXRD patterns shown in Fig. S8 confirms that the phase of the investigated bulk material is the same as that of the reported single structure determination.

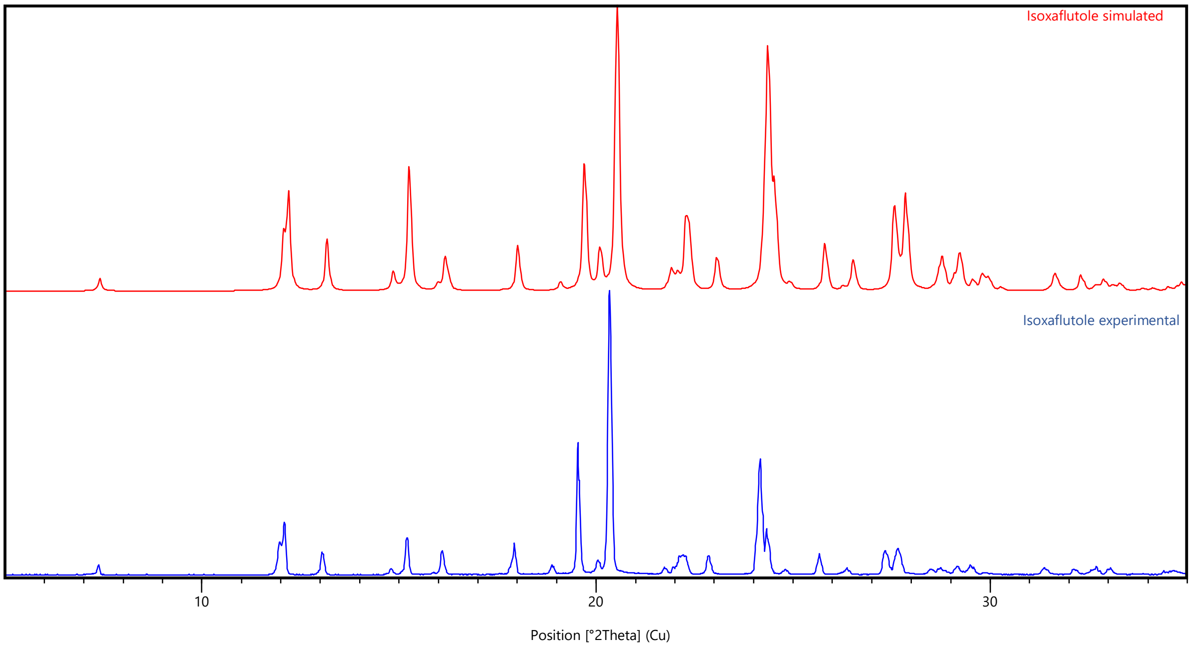

Fig. S8. Top: PXRD diagram simulated from the single crystal structure data of (I) (193 K; Cu radiation, λ = 1.54056 Å). Bottom: experimental PXRD pattern (298 K).

Experimental: The PXRD patterns were obtained with an X'Pert PRO diffractometer (PANalytical, Almelo, The Netherlands) equipped with a θ/θ coupled goniometer in transmission geometry, programmable XYZ stage with well plate holder, Cu-K$\alpha_{1,2}$ radiation source with a focusing mirror, a 0.5° divergence slit, a 0.02° soller slit collimator and a 0.5° anti-scattering slit on the incident beam side, a 2 mm anti-scattering slit, a 0.02° soller slit collimator, a Ni-filter and a solid state PIXcel^{1D} detector. The patterns were recorded at a tube voltage of 40 kV, tube current of 40 mA, applying a step-size of 0.013° 2θ with 40 s per step in the angular range of 2° to 40°.

8. References

1 a) U.S. Environmental Protection Agency, *PhysProp database. Estimation Programs Interface Suite™ for Microsoft® Windows, v 4.11: Isoxaflutole (CASRN 141112-29-0)*, **2012**; b) Jean-Claude Bradley, Antony Williams, Andrew Lang, *Jean-Claude Bradley Open Melting Point Dataset*, figshare, **2014**.

2 J. Rouchaud, O. Neus, H. Eelen, R. Bulcke, *Archives of environmental contamination and toxicology* **2002**, *42*, 280.
